# Remedy for Croup

**Published:** 1877-07

**Authors:** 


DR. TEFFT, of Topeka, Kansas, writes that he has never failed to cure a case of croup, in thirty-five years practice, with simple ice or ice water. He goes on to say that the membrane cannot form without heat, and when it is formed if the parts are kept absolutely cool it will fall off and be discharged.

